# Pembrolizumab and tavokinogene telseplasmid electroporation in metastatic melanoma

**DOI:** 10.1016/j.ijscr.2020.11.063

**Published:** 2020-11-16

**Authors:** Yonatan Dollin, Jason Rubin, Richard D. Carvajal, Helene Rached, James R. Nitzkorski

**Affiliations:** a Department of Medicine, Wright State University, USA; b Department of Medical Oncology, Vassar Brothers Medical Center, Poughkeepsie, NY, USA; c Department of Medicine, Columbia University Medical Center, New York, NY, USA; d Department of Surgery, Vassar Brothers Medical Center, USA

**Keywords:** Immunotherapy, Melanoma, Tavokinogene telseplasmid, Electroporation, IL-12

## Abstract

**Introduction:**

Tavokinogene Telseplasmid Electroporation Therapy (TAVO) and Pembrolizumab therapy is being studied in subjects with immune checkpoint inhibitor (ICI) resistant melanoma. TAVO is a novel office-based local therapy shown to be effective in patients with advanced melanoma. The technique involves the direct injection of a plasmid encoding IL-12 into an accessible tumor driven by electroporation. The tumor cells have then been shown to express high levels of IL-12 resulting in a local inflammatory response within the tumor microenvironment.

**Presentation of case:**

The patient with stage IIB, pT3b melanoma was treated with primary tumor resection and found to have a negative sentinel node biopsy. She subsequently developed regional recurrence and was treated with inguinal lymphadenectomy and adjuvant Nivolumab. Despite therapy, she had progression of disease with skin and subcutaneous metastases (in-transit lesions), brain and liver lesions, hilar and iliac nodal disease. She was transitioned to nivolumab + ipilimumab, and Talimogene Laherparepvec (T-VEC) therapy for the in-transit lesions, without success. Stereotactic radiosurgery was used for the brain metastasis. Groin subcutaneous and in-transit lesions were treated with TAVO and intravenous pembrolizumab. Serial physical exams and CT scans were used to assess response.

**Discussion:**

All lesions treated with TAVO resolved. An abscopal response was also noted: hilar and mediastinal lymphadenopathy resolved. The liver mass and pelvic lymphadenopathy decreased in size, and her brain metastasis remained stable after radiation.

**Conclusion:**

This case suggests that combination TAVO and Pembrolizumab is a safe and effective local treatment for ICI resistant metastatic melanoma in the setting of rheumatoid arthritis. An abscopal effect was also noted through control of systemic disease.

## Highlights

• TAVO is a novel office-based local therapy effective in patients with advanced melanoma.
• Involves direct injection of an IL-12 plasmid into an accessible tumor by electroporation.
• Case study to assess efficacy of TAVO in patients with rheumatoid arthritis.
• Found to be a safe and effective local treatment with abscopal effect.

## 1 Introduction

### 1.1 IL12 treatment for cancer

IL-12 is an inflammatory cytokine expressed by dendritic cells, macrophages, and neutrophils [1] that activates helper and cytotoxic T-cells [2]. The main anti-tumor properties of IL-12 include: inhibiting angiogenesis, increasing the activation and survival of memory T-cells allowing for adaptive immunity, and inhibiting T regulatory cells and TH2 cells. TH2 cells are involved in one of the key primary tumor immune evasion pathways [[3], [4], [5]]. Similar to other inflammatory cytokines like high dose IL-2 and IFN-α-2a, systemic exogenous treatment of tumors with IL-12 has been shown to be highly toxic and ineffective [6]. When injected locally into solid tumors, exogenous IL-12 has been shown to induce a switch from predominance of Th2 cells to Th1 cells and a partial local response. However, IL-12 continues to cause toxicity, as well as a lack of activation of an effective systemic immune response [6].

### 1.2 Tavokinogene telseplasmid (TAVO) therapy

TAVO therapy, (Oncosec, Pennington, NJ), is a novel therapy in combination with pembrolizumab for patients with advanced melanoma, currently under clinical trial. The technique involves the direct injection of a plasmid encoding IL-12 into an accessible tumor. The site is then treated with electroporation to induce tumor cell expression of IL-12 resulting in a local inflammatory response in the tumor microenvironment [6]. In addition to causing a local response, TAVO’s previous clinical trials have demonstrated an abscopal effect, with some patients experiencing a systemic response after local therapy [6].

## 2 Case presentation

We present a 58-year-old female with a history of rheumatoid arthritis, off medication, and melanoma of the left lateral foot who developed recurrence and disease progression after surgery. Family history is significant for mother with melanoma and colon cancer. This case report has been reported in line with the SCARE 2018 criteria [11].

Her clinical course began with wide excision of the skin and subcutaneous tissue of the left lateral foot primary with negative margins and a sentinel node biopsy; Stage IIB pT3aN0M0. The DecisionDx-Melanoma™ 31 gene panel (Castle Biosciences, Friendswood, TX) demonstrated a high risk of five-year recurrence. No BRAF, KIT, NRAS mutations were identified. She developed an inguinal recurrence after a short disease-free interval (3mo). After full staging investigations which were negative for pelvic or distant metastatic disease, she underwent a left superficial and deep inguinal lymphadenectomy with sartorius muscle transposition flap followed by nivolumab therapy, which was well tolerated.

She subsequently developed visible cutaneous in-transit lesions in the left groin concerning for recurrence. Restaging with CT and MRI scans confirmed new brain and liver (1.7 cm) lesions and the presence of hilar and pelvic nodal disease. Secondary to her rheumatoid arthritis, the patient was not eligible for clinical trials. Ipilimumab in combination with nivolumab was attempted without success. Talimogene Laherparepvec (T-VEC) therapy was attempted for severely symptomatic in-transit lesions without success. Stereotactic radiosurgery was used to control her brain metastasis, and external beam radiation was used to treat her in-transit lesions and pelvic adenopathy with a partial response.

FDA approval for expanded access on a compassionate use basis of a novel therapeutic treatment TAVO + Pembrolizumab was obtained (FDA IND 18672). This was then approved by our institutional IRB (2018–17). Informed consent was obtained from the patient. Two in-transit lesions in the left groin were treated by direct injection of TAVO and electroporation. This was given in concert with intravenous pembrolizumab. TAVO Treatment cycles on days 1, 5, and 8 were given every six weeks, and pembrolizumab was given every three weeks.

## 3 Results

**Fig. 1:**
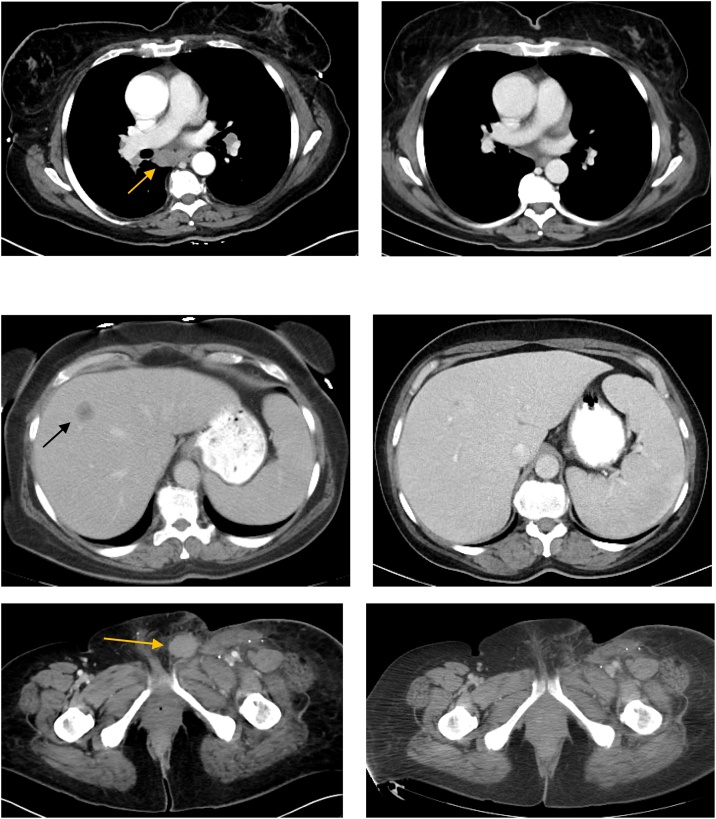
Response to Combination Pembrolizumab and Tavokinogene Telseplasmid (TAVO) Electroporation Therapy. A. Hilar lymphadenopathy before and after therapy. B. Liver metastasis before and after therapy. C. Left groin target lesion was the lesion used for treatment, seen before and after therapy.

After the third treatment cycle, the treated in-transit lesions resolved. Her brain lesion was stable with no new findings or local recurrence. The hilar and mediastinal lymphadenopathy resolved, her pelvic nodes decreased in size and her liver mass regressed from 1.7 cm to 6 mm (Fig. 1). In the absence of an accessible lesion amenable to TAVO, it was stopped and she was maintained on pembrolizumab alone. Approximately 12 months after her response, surveillance CT scan demonstrated a new contralateral deep pelvic adenopathy meeting criteria for progression of disease. The new disease, however, cannot be reached with the probe and as such cannot be treated with TAVO. Other investigative options are being pursued.

## 4 Discussion

The evaluation of TAVO therapy has been an ongoing process with two completed clinical trials for drug safety (phase I and phase II) and a third phase II trial in progress studying combination therapy with Pembrolizumab.

The initial phase I, dose escalation clinical trial evaluated efficacy, tolerability, and toxicity of the TAVO therapy. The trial involved twenty-four patients in seven cohorts who were treated with differing drug concentrations between 0.1–1.6 mg/mL in lesions located in differing areas of the body [7]. Nineteen out of twenty-four had metastatic lesions. Out of these 19, ten (53%) showed objective regression or stable disease. In three of the patients (15%), all distant lesions resolved [7]. This trial demonstrated an abscopal effect of the TAVO therapy as well as the effectiveness of electroporation plasmid delivery system as a cancer therapeutic.

A confirmatory phase II multicenter clinical trial was performed in fifty-one patients [8]. One treatment cycle consisted of TAVO treatment on days 1, 5, 8 in up to four lesions [9]. ORR (objective response rate) was 33% (9/27) with a CR (complete regression) of 11% (3/27) in the treated lesions [9]. They observed regression of non-injected lesions in 62% (13/21) of patients [9]. On biopsy, the number of intra-tumoral NK cells were doubled with a noted increase in activated NK cells in the patient’s serum [9]. This trial confirmed the abscopal effect of TAVO and provided data supporting the inflammatory mechanism behind the drug’s efficacy.

The in-progress phase II clinical trial is evaluating the TAVO + Pembrolizumab treatment efficacy in Stage III/IV melanoma patients who have disease progression on anti-PD-1 agents (Pembrolizumab and Nivolumab) [10]. We were unable to enroll our patient in this study due to her rheumatoid arthritis.

In our case study, the patient with Stage IIB melanoma had disease progression despite treatment with anti-PD-1 therapy and failed Nivolumab and Nivolumab with Ipilimumab. With TAVO + Pembrolizumab, she showed a complete response in her treated lesions, as well as a response in her distant lesions. The patient was not receiving treatment for her rheumatoid arthritis and thus a good response to treatment was observed. The adverse events included: patient reported tenderness at the injection site with cellulitis, that responded to antibiotics. No exacerbation of her rheumatoid arthritis symptoms were observed. In all the patient was compliant with treatment and satisfied with her treatment conditions and response.

## 5 Conclusion

This demonstrates one case of TAVO sensitizing the patient’s checkpoint inhibitor refractory disease to Pembrolizumab despite the complicating factor of rheumatoid arthritis. This case suggests that autoimmune disease is not necessarily an excluding factor in effective TAVO treatment in patients that fail checkpoint inhibitors and thus further study is warranted.

## Declaration of Competing Interest

James Nitzkorski is a consultant for Oncosec. Richard D. Carvajal is a consultant for AstraZeneca, Bristol-Myers Squibb, Castle Biosciences, Foundation Medicine, Immunocore, Incyte Merck, Novartis, Roche/Genentech, Aura Biosciences, Chimeron, and Rgenix.

## Sources of funding

This research did not receive any specific grant from funding agencies in the public, commercial, or not-for-profit sectors. TAVO therapy and materials was provided without cost by Oncosec, Pennington, NJ.

## Ethical approval

FDA approval for expanded access use of a novel therapeutic treatment TAVO + Pembrolizumab was obtained FDA (IND 18672), was approved by our institutional IRB (2018–17), and informed consent was obtained from the patient.

## Consent

Written informed consent was obtained from the patient for publication of this case report and accompanying images. A copy of the written consent is available for review by the Editor-in-Chief of this journal on request.

## Authors contribution

Yonatan Dollin prepared the manuscript, James Nitzkorski conducted, oversaw, and drove the research and edited the manuscript, Richard Carvajal and Jason Rubin both advised and edited the manuscript. Helene Rached edited and submitted the manuscript.

## Registration of research studies

1. Name of the registry: ClinicalTrials.gov
2. Unique identifying number or registration ID: NCT03132675, NCT01502293
3. Hyperlink to your specific registration (must be publicly accessible and will be checked): https://www.clinicaltrials.gov/ct2/show/NCT03132675 https://www.clinicaltrials.gov/ct2/show/NCT01502293?term=electroporation+il-12&draw=1

## Guarantor

James R. Nitzkorski, MD.

## Provenance and peer review

Not commissioned, externally peer-reviewed.

## Special conference consideration

The authors deeply appreciate being given the opportunity to present this abstract at the Academic Surgical Conference, Orlando FL, Feb 2020.
